# A Trianalyte µPAD for Simultaneous Determination of Iron, Zinc, and Manganese Ions

**DOI:** 10.3390/molecules29204805

**Published:** 2024-10-11

**Authors:** Barbara Rozbicka, Robert Koncki, Marta Fiedoruk-Pogrebniak

**Affiliations:** Faculty of Chemistry, University of Warsaw, Pasteura 1, 02-T093 Warsaw, Polandrkoncki@chem.uw.edu.pl (R.K.)

**Keywords:** iron, zinc, manganese, trianalyte µPAD

## Abstract

In this work, a microfluidic paper-based analytical device (µPAD) for simultaneous detection of Fe, Zn, and Mn ions using immobilized chromogenic reagents Ferene S, xylenol orange, and 1-(2-pyridylazo)-2-naphthol, respectively, is presented. As the effective recognition of analytes via respective chromogens takes place under extremely different pH conditions, experiments reported in this publication are focused on optimization of the µPAD architecture allowing for the elimination of potential cross effects. The paper-based microfluidic device was fabricated using low-cost and well-reproducible wax-printing technology. For optical detection of color changes, an ordinary office scanner and self-made RGB-data processing program were applied. Optimized and stable over time, µPADs allow fast, selective, and reproducible multianalyte determinations at submillimolar levels of respective heavy metal ions, which was confirmed by results of the analysis of solutions mimicking real samples of wastewater. The presented concept of simultaneous determination of different analytes that required extremely different conditions for detection can be useful for the development of other multianalyte microfluidic paper-based devices in the µPAD format.

## 1. Introduction

A continuous technological development is inextricably linked to the increased release of so-called heavy metals into the environment. Through water, soil, and air, these metals get into food products and then into organisms. All this ultimately leads to environmental pollution. Many metal ions have a possibility of binding to vital cellular components what make them easier to bioaccumulate. The result is a growing number of people whose elevated levels of heavy metals in their bodies disrupt physiological processes and even cause poisoning [1,2,3]. As in the production processes, several metal ions may be removed at the same time; it is crucial to develop analytical systems for simultaneous detection of more than one analyte. Moreover, the multiplex devices should be easy to fabricate, easy to use, fast, cheap, reliable, and portable. Furthermore, it would be desirable if the proposed devices could work outside of lab conditions in the outside lab conditions.

The paper-based analytical devices fulfill all these features as the paper is available all over the world, and it is easy to modify. As a result, the geometry of channels and zones depends only on the needs and desired application, and the analyst’s imagination is the limit. Among microfluidic paper-based analytical devices (µPADs), the number of articles dealing with parallel determination of multiple analytes is increasing [4,5,6,7,8]. The multianalyte systems have been developed for the simultaneous detection of several metal ions in wastewater samples [5], real water samples [6,7], future use in environmental and biomedical analyses [8], etc.

Multianalyte detection is also often necessary for biomedical analyses, where elevated metal ion levels might affect the final diagnosis. Iron, zinc, and manganese ions are important metal ions in production processes. On the other hand, all these ions affect the level of cholesterol in organisms [9,10,11,12]. Their non-physiological levels might cause elevated cholesterol (especially the so-called “bad cholesterol” level) in blood. In the case of biomedical analyses, the developed analytical systems should be also easily disposable as the used samples are potentially infectious. This can be provided by paper-based analytical devices because burning the devices is the easiest disposal method.

The most popular detection method in µPADs is colorimetry as it can be used without any instrumentation (naked eye detection), with semi-quantitative analysis, or be combined with a scanner or smartphone portable detectors for quantitative analyses. The iron ions may be detected colorimetrically using 1,10-phenanthroline or Ferene S. With those chromogenic reagents, iron ions form orange–red and green–blue complexes, respectively [13,14]. For zinc ions, detection with xylenol orange, with which the Zn^2+^ forms an intensively red-colored complex [15,16,17], or dithizone, forming a red–pink product [6], may be utilized, whereas manganese ions may be detected using the most popular reagent—4-(2-pyridylazo)resorcinol (PAR) [18,19].

In this article, the simple microfluidic paper-based analytical device for simultaneous colorimetric detection of three analytes (iron(II), zinc(II), and manganese(II) ions) is proposed. The common problem in this kind of multianalyte microfluidic system is that one reaction needs to be conducted in an acidic pH, whereas another requires an alkaline pH. In this article, the simple solution of this problem is proposed. Three metal ions are detected with three detection zones which are adequately separated. Moreover, a problem with metal ion transport across the filter paper as well as a problem with a reagent leakage were eliminated. The work presents the concept of using non-water-soluble chromogen immobilized in µPAD as the idea for increasing the sensitivity and repeatability of detection.

## 2. Results and Discussion

### 2.1. Paper-Based Detection Zones

**Figure 1:**
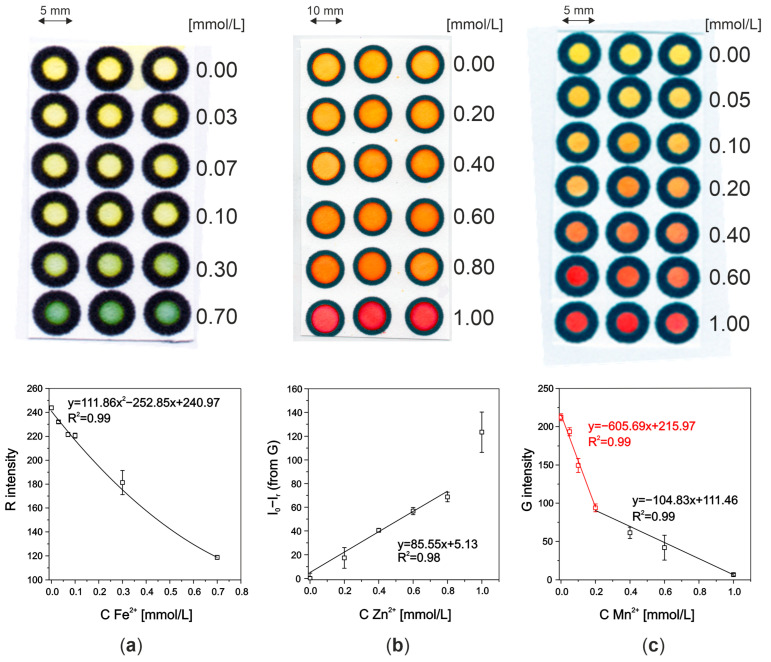
Detection zones after colorful detection reactions for (**a**) iron(II), (**b**) zinc(II), and (**c**) manganese(II) ions (top) and corresponding calibration graphs (bottom).

The detection zones for each analyte were optimized separately to find the most favorable parameters before combining them into a multianalyte system. Firstly, experiments were conducted using detection zones of 10 mm diameter. Exemplary photos of the detection zones are presented in Figure 1.

The optimization process included the same parameters for each ion. Among the tested ones, the filter paper type, the concentration of dyes, the dyes’ air drying time, and the diameter of the detection zones were studied. Regarding the filter paper type, the Whatman papers no. 1–6 were studied. For all three ions, type no. 1 was chosen as the preferred one ensuring optimal flow rate and acceptable repeatability of the measured signals. This means that the whole µPAD for the three ions’ detection can be fabricated using one paper type.

For every ion, the detection zones of 5, 7, and 10 mm diameter were studied. In each case, the width of the borders of the zones was 1 mm (before heating). Moreover, 1 µL of dye solutions and then standards were applied successively to 5 mm diameter detection zones, while 5 µL and 8 µL were applied to 7 mm and 10 mm diameter circles, respectively. As the last parameter studied, the geometry of channels was examined. Furthermore, while designing the simple microfluidic systems, different geometries of channels connecting sample and detection zones were examined—length × width: 20 × 3, 20 × 2, 15 × 2, 15 × 3, 10 × 2, and 10 × 3 mm.

#### 2.1.1. Iron Ions

Ferren S has a yellow color, and it forms a blue complex with iron(II) ions—for lower concentrations of iron(II), a yellow–green or green color appears on the paper (see Figure 1a). For choosing the optimal concentration of Ferene S for iron ion detection, solutions of 1–90 mmol/L were tested (Figure S1a in Supplementary Materials). Among the studied ones, the 60 mmol/L and 90 mmol/L solutions of Ferene S were the most promising; so, only these two concentrations were examined for smaller concentrations of iron ions (Figure S1b). The concentration of 90 mmol/L was selected for further experiments as the results were characterized by the highest sensitivity and good linearity of the response as well as better stability of the obtained color after reaction.

The chromogenic reagent was deposited onto the detection zone prior to the standard solutions. The times of 15, 30, and 60 min of dye air drying time were studied, and the time of 15 min was chosen as the optimal one. However, no significant differences observed in the analytical parameters between dye drying times were observed which may be useful in the future for preparation and storage of ready-to-use paper strips.

After experimental selection of detection zone diameters, the best sensitivity and linear response was obtained for the 5 mm detection zone, and this size was used for further research. Corresponding calibration curves for choosing optimal parameters are presented in the Supplementary Materials (Figure S1).

After the experimental examination of all given geometries of the microfluidic system, for iron ion detection, the chosen sample zone has a diameter of 10 mm, whereas the detection zone has a 5 mm diameter. For each microfluidic system, a different calibration curve was obtained, both in terms of sensitivity and the received R^2^ (Figure S2). At this step, the optimal geometry of the channel was not chosen.

#### 2.1.2. Zinc Ions

Optimal parameters for zinc(II) ion detection were chosen experimentally. For these experiments, xylenol orange (forming with Zn(II) a complex of a molar ratio of 1:1) dissolved in acetate buffer (pH = 4.4) was used throughout [15,16,17].

Different air-drying times were studied—15 min, 2 h, and 24 h. The best sensitivity was obtained for a 15 min drying time. Then, the optimal detection zone’s diameter was selected. Similar sensitivities and linearities were received for both—5 and 10 mm—detection zones. These two zones were examined in the simple microfluidic systems containing a sample zone, channel, and two sizes of detection zones (5 and 10 mm diameter). While applying only 1 layer of xylenol orange in the microfluidic systems, there was a problem with the dye flowing to the edge of the detection zone This phenomenon was not very repeatable and affected the linearity of the determination. To improve this, several layers of dye were applied to the detection zone (5 and 10 mm diameter detection zones were studied). Different concentrations (2, 5, and 10 mmol/L) and 1–3 layers of dye—xylenol orange—were examined (Figure S3). Each layer was deposited on the filter paper after full air drying of the previous layer. The best sensitivity and linearity were obtained for 5 mmol/L dye added to the 10 mm diameter detection zone as 2 layers. As a result, this type of Zn-detection zone was used in further research.

#### 2.1.3. Manganese Ions

The concentration of PAR was selected experimentally among 0.014, 0.14, 0.28, 0.7, 2.8, 7, and 14 mmol/L (data presented in Figure S4, Supplementary Material). The optimal one was 2.8 mmol/L, giving the highest sensitivity in comparison to other PAR solutions. Moreover, the lower concentration of PAR means the method is more environmentally friendly. Different time gaps between standards deposition and detection were studied: 0, 15, 30, and 60 min. The optimal ones were conducting detection immediately or after 15 min. Moreover, 5, 7, and 10 mm detection zone diameters were also tested. The lowest diameter gave the best sensitivity and was chosen for further experimental work. The optimal pH (pH = 12) for Mn(II) ions’ colorful reaction was used at the detection zone as it was recommended elsewhere [18].

**Figure 2:**
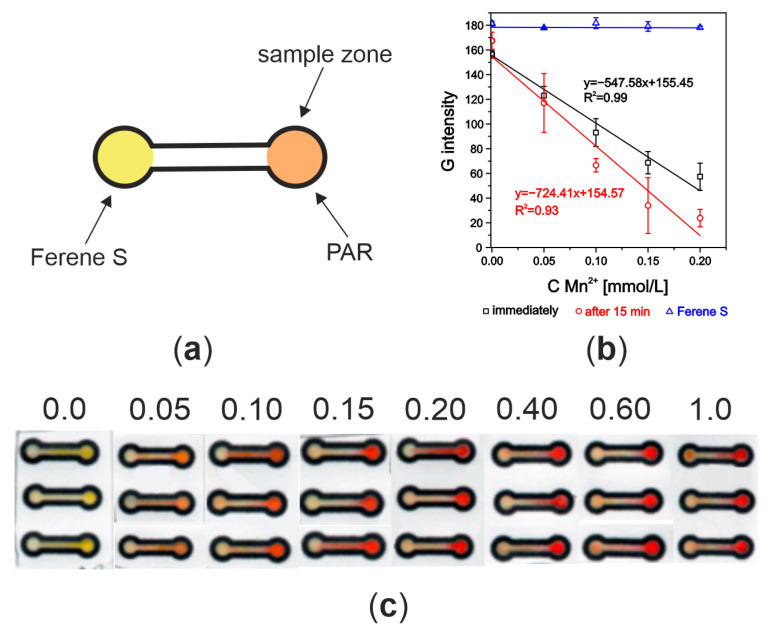
(**a**) µPAD shape with two detection zones; (**b**) calibration curves after adding standard solutions of Mn^2+^ to the detection zone with PAR immobilized; (**c**) photo of the tested µPADs (concentrations of Mn^2+^ are given in [mmol/L]).

Different geometries of channels were examined without receiving any positive results (no linearity in calibration curve and poor signals). The detection zone was not colored as it was while depositing the standards straightway on the detection zone. It looked like manganese ions do not migrate in the filter paper from the sample zone to the detection zone. To solve this problem, it was checked if the detection zone of manganese ions could be at the same place as the sample zone. The shape of the µPAD used in this experiment is presented in Figure 2a. The yellow circle is the Fe detection zone, where the Ferene S (1 µL) was deposited, whereas the orange circle is the Mn detection zone, where the PAR solution (1 µL) was deposited. After 15 min of air drying of chromogenic reagents, 10 µL of manganese(II) ion standards were added to the sample zone. In the photo (Figure 2c), it is seen how the PAR was transported with the added standard solution—the orange–red color is also visible in the channel. The Fe detection zone’s color was a little bit changed in spite of there being no iron ions in the samples. Although there was almost no change in signal on the Ferene S detection zone, the PAR moving into the second zone may affect future iron ion determination. In Figure 2b, the calibration curves obtained after the Mn^2+^-PAR reaction was scanned immediately after the solution reached both detection zones and after 15 min are shown. Additionally, the color changes for the detection zone where Ferene S was deposited are presented (Figure 2c).

### 2.2. Construction of Paper-Based Microfluidic System

In the bianalyte microfluidic system, two detection zones were combined together—for iron(II) and manganese(II) ion detection. The system in the first version looked like the one presented in Figure 2a. Moreover, 15 min after depositing the dyes, the standard solutions containing Mn(II), Fe(II), or their mixture were applied. Detection was carried out after the standard solutions reached the Fe detection zone. The green color intensities for the same concentrations of manganese ions in standards with and without Fe(II) ions differ significantly. This is caused by the pH change—iron(II) standards are acidified with ascorbic acid (to reduce iron(III) to iron(II)). Moreover, the color reaction of Fe(II)-Ferene S takes place at pH ca. 2, so the detection zone with immobilized Ferene S is acidified additionally with HCl solution. On the other hand, the pH of the Mn detection zone should be alkaline (ca pH = 12). After the standards were deposited and reached both detection zones, the pH of both detection zones was changing—it increased for the Fe zone and decreased for the Mn zone. As a result, it was impossible to determine both manganese and iron ions. For manganese, the obtained signal was not repeatable because of non-reproducible pH changes and PAR flow in each µPAD. The characteristic color of the Ferene S complex with iron ions was also not observed on µPADs because PAR, together with the standards, moved to the zone containing Ferene S, changing the color of the zone, what masks the true color of the reaction with iron(II) ions.

To avoid the influence of acidification of the Mn detection zone by the standards, the PAR detection zones were prepared separately to the rest of the microfluidic system (channel and Fe detection zone). The pieces of filter paper (3 × 1 cm) were dipped into the PAR solution, then air dried, and afterwards were cut into circles of 4 mm diameter using a punch.

**Figure 3:**
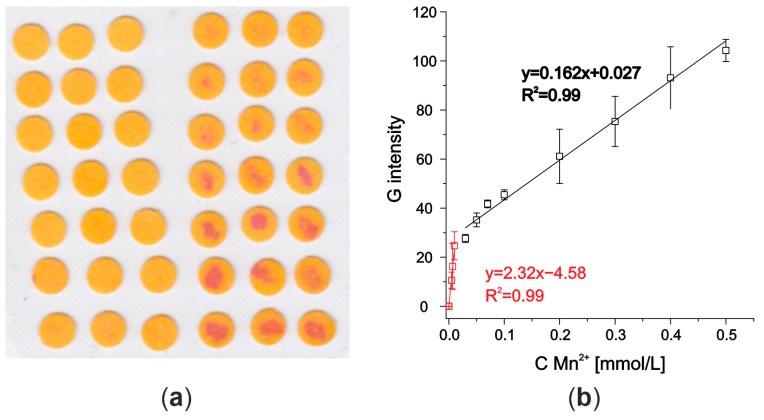
(**a**) Scanned detection zones with PAN immobilized after Mn^2+^ standards deposition; (**b**) corresponding calibration curve with two linear ranges.

Moreover, a dye similar to PAR—(1-(2-pyridylazo)-2-naphthol (PAN)—was tested as it reacts in a similar way with manganese (see Figure 3) and other metal ions. However, since it does not dissolve in water, its leaching together with the standard solution could be limited. The circle zones with PAN were prepared also by immersing filter paper pieces into the PAN solution (1.25 g/L in THF). As the complex formation is more efficient in an alkaline environment [20,21], the Mn detection zones were alkalized by bathing filter paper pieces in NaOH solution of pH = 12 and 13 for 1 min each. Then, all papers were air dried and detection zones were cut in the shape of circles (d = 4 mm) using a punch.

**Figure 4:**
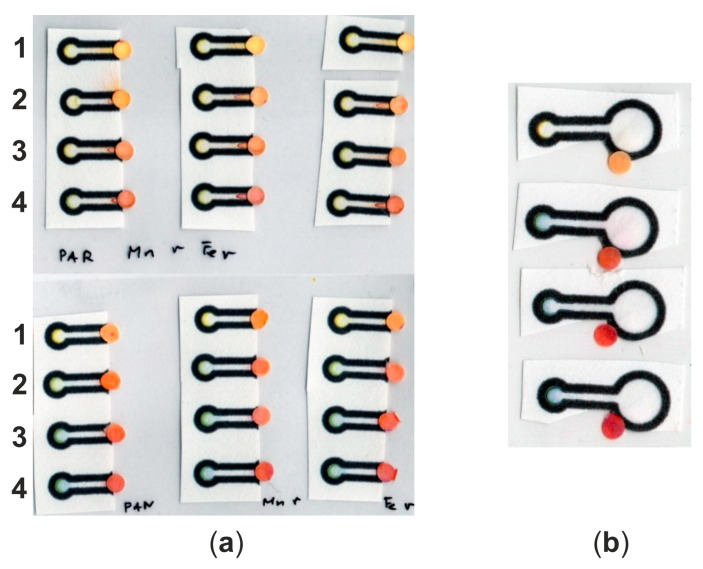
(**a**) Comparison of the µPADs with PAR (**top**) and PAN (**bottom**) at Mn detection zones; each standard was tested three times (3 consecutive repetitions of the same standard in rows); on the other side of the detection zone, Ferene S was deposited; (**b**) the second configuration of a bianalyte µPAD with Mn and Fe detection zones and a sample division zone. Standards concentrations used for these experiments are given in Table 1.

To form a bianalyte microfluidic system, two pieces of the system—the printed part (channel and Fe detection zone) and a cut Mn detection zone—were glued on the cold laminating pouches to avoid elements from moving (see Figure 4).

**Table 1:** Concentration of standards used presented in Figure 4 in rows.

Standard No.	Fe^2+^ [mmol/L]	Mn^2+^ [mmol/L]
1	0.00	0
2	0.05	0.050
3	0.10	0.075
4	0.15	0.100

As can be seen from Figure 4a (top), using PAR as a chromogenic reagent for Mn(II) detection even at separate detection zones causes migration of the dye into the channel. The applied approach (separation and alkalization) ensures visible color change in the Mn detection zone with increased Mn(II) concentration. Nevertheless, there was no color change at the Fe detection zone in all cases (with increasing Fe(II) concentration). On the other hand, no migration of dye is observed when PAN is used as a chromogenic reagent (Figure 4a, bottom). What is more, the color change is visible with the naked eye at both detection zones.

However, the use of NaOH solutions (pH = 12 and 13) did not improve the determination of manganese ions—still, the repeatability of signals as well as the linearity was not satisfactory. The pH of the Mn detection zone after deposition of the standards was around 6, which is too low to form an Mn-PAN complex. To increase the pH at the Mn detection zone, higher NaOH concentrations (from 0.1 to 1 mol/L) as bath solutions were tested. The paper pieces after immersing in the PAN solution were bathed in NaOH solutions. Using NaOH concentrations from 0.4 to 1 mol/L caused remaining the filter papers red even after drying (filter papers bathed in NaOH solutions of lower concentrations after drying return to yellow again). The already red-colored Mn detection zones after the NaOH bath were useless in the context of detection of a red-colored Mn-PAN complex. Moreover, high NaOH concentrations in the sample zone caused an increase in pH in the Ferene S zone, which made it impossible to determine iron(II) on such µPADs.

A breakthrough in this research was achieved by changing the angle between the sample/Mn detection zone and the Fe detection zone and using a separated sample division zone (see Figure 4b). Finally, to fabricate the bianalyte system, the Fe detection zone was acidified by applying 1 µL of 0.01 mol/L HCl solution, air drying, and deposition of 1 µL of Ferene S solution. The Mn detection zone was firstly immersed in PAN solution, air dried, bathed for 1 min in 0.3 mol/L NaOH solution, and then air dried, and finally cut. The resulting calibration curves are presented in Figure S5. The angle in the trianalyte system between the Mn and Fe detection zones should be optimized to ensure proper pHs for both colorful reactions.

### 2.3. Analytical Performance of Paper-Based Trianalyte System

**Figure 5:**
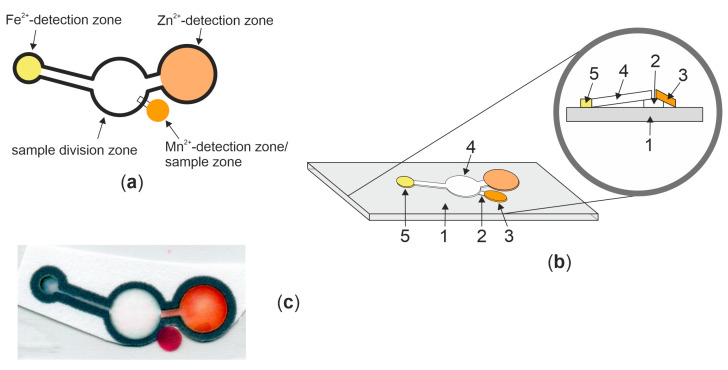
(**a**) µPAD shape with three detection zones and a sample division zone; (**b**) layer by layer view of the designed µPAD; (**c**) the photo of the designed µPAD (after the reaction with all ions. Numbers refer to the following: 1—cold laminating foil, 2—connector between sample zone and sample division zone, 3—Mn^2+^ detection zone/sample zone, 4—sample division zone, 5—Fe^2+^ detection zone.

The trianalyte microfluidic paper-based analytical devices contain three detection zones, among which one plays the role of the sample deposition zone. All these zones were connected by the sample division zone (see Figure 5).

The Fe zone consists of a 5 mm detection area (acidified with 1 µL 0.01 mol/L HCl solution and, after drying, modified with 1 µL of 90 mmol/L Ferene S solution) connected with the sample division zone a the channel of 10 mm in length and 2 mm in width. The Zn part contains a 10 mm detection zone with two layers of 5 mmol/L xylenol orange in acetate buffer. This zone was connected with the middle zone via the 3 mm channel of 3 mm width. The Mn detection zone (sample zone as well) is the circle of 4 mm diameter with PAN immobilized and alkalized with 0.3 mol/L NaOH. It is connected with the sample division zone via the channel that is 3 mm in length and 1 mm in width.

The trianalyte µPADs were tested using standards containing increasing or constant concentrations of all ions, while the pH of the blank was identical to the pH of the standards. The results of determination of iron(II), zinc, and manganese(II) ions in different configurations of analyte concentrations are presented in the Supplementary Materials (Figure S6). For each measurement, 36 µL of standards/samples were deposited on the sample zone. In the graphs presented in Figure S6 are the comparisons of calibration curves and their sensitivities obtained for combinations with all ions at variable concentrations with the results for standards in which there was an increasing concentration of only the ion determined on a given dye. The obtained sensitivities differ slightly which means that the three analytes—Fe(II), Zn(II), and Mn(II)—do not affect each other’s determinations.

**Figure 6:**
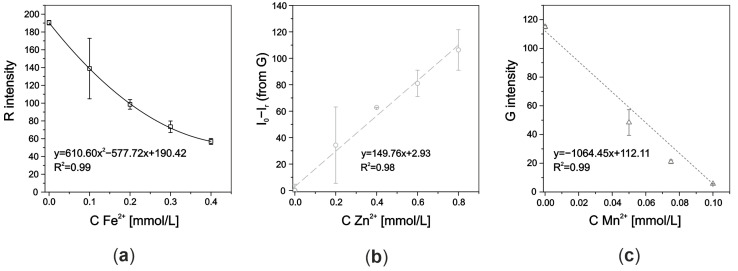
Calibration curves obtained using a trianalyte µPAD for (**a**) Fe^2+^, (**b**) Zn^2+^, and (**c**) Mn^2+^ ions using standards containing a mix of the three ions.

The calibration curves obtained for each analyte (using standards containing increasing concentrations of each ion) are presented in Figure 6.

The developed trianalyte µPAD was characterized by several analytical parameters such as the limit of detection, limit of quantification, accuracy, and precision. The obtained parameters are presented in Table 2. The LOD and LOQ were calculated as a value of an average signal of blank plus three or six times the values of the standard deviation, respectively (see Equations (1) and (2)).

(1) $$LOD=y_{aver}+3\cdot SD$$

(2) $$LOQ=y_{aver}+6\cdot SD$$

Accuracy was calculated for specific concentrations of analytes in the sample containing 0.800, 0.125, and 0.400 mmol/L for Zn(II), Mn(II), and Fe(II), respectively. Precision was calculated as the RSD [%] of the measured signals for the same sample. The interday reproducibility values were 5.2%, 3.6%, and 1.9% for Fe^2+^, Zn^2+^, and Mn^2+^, respectively. Moreover, analytical parameters of the systems described in the literature dedicated to the determination of Fe(II), Zn(II), and Mn(II) are presented in Table 3. In Table 3, the developed µPAD is also added to make it easier to compare.

As can be seen from Table 3, the obtained limits of detection as well as the analytical ranges are comparable or even better than those presented in the literature. Moreover, the cited articles, besides [5] where five ions were detected, are dedicated to determination of only a single analyte. However, the paper test described in [5] consists of five separated detection zones which is similar to the circles presented in this work, as seen in Figure 1. It requires sample delivery to each zone separately. Moreover, using separate detection zones means that there is no problem of different environmental conditions being required in each zone. In this work, the final device consists of three detection zones which are connected by microchannels which simplify the sample deposition. Furthermore, the appropriate geometry of the system ensures that the detection conditions do not influence one another despite the connection between them.

The developed µPADs were examined over time in two different storage conditions. The fully prepared devices (printed, cut, modified with reagents, and glued on the cold laminating pouches) were stored in foil at room temperature and at 4 °C (fridge). Stability studies were conducted for 2 weeks. The ready-to-use µPADs stored in the fridge were left for several minutes to reach room temperature before deposition of standard solutions.

**Figure 7:**
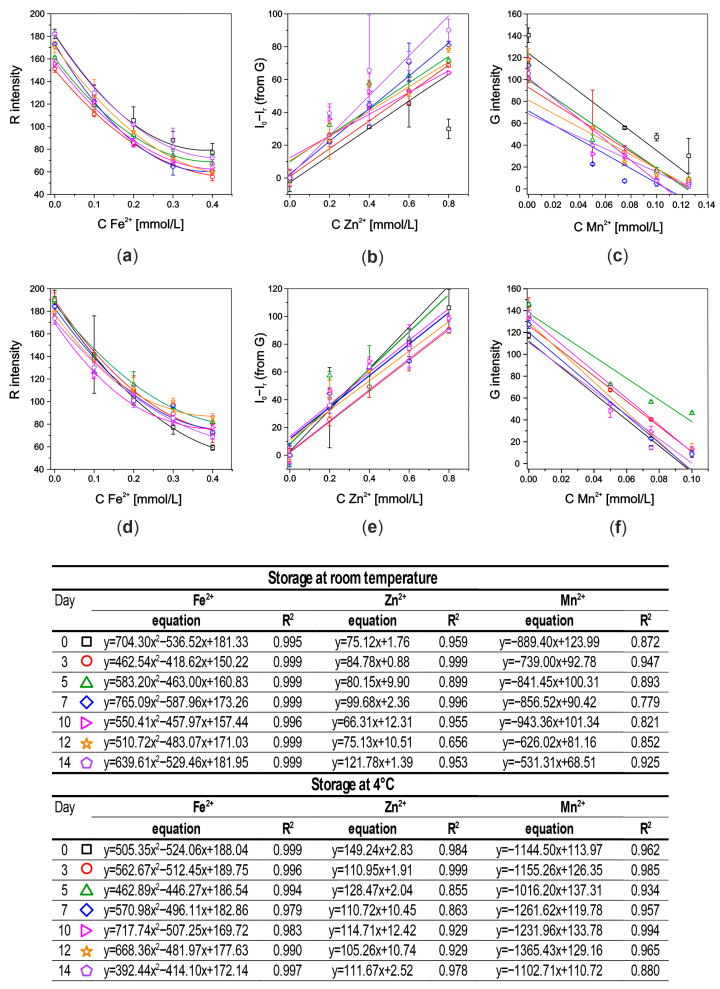
The obtained calibration curves using systems stored for two weeks at room temperature (**a**–**c**) and at 4 °C (**d**–**f**) for (**a**,**d**) Fe^2+^, (**b**,**e**) Zn^2+^, and (**c**,**f**) Mn^2+^ ions. Below are the obtained equations and R^2^ for each calibration curve.

When testing systems stored at room temperature, a deterioration in the sensitivity and linearity of the assays was observed, especially for the Mn and Fe detection zones. The obtained calibration curves using systems stored at room temperature and in the fridge are shown in Figure 7.

As can be seen from the graphs presented in Figure 7, the developed microfluidic paper-based device for simultaneous determination of three ions is stable for a minimum of 2 weeks while stored in the fridge. The RSD of the slope comparison between days during these 2 weeks was between 9.4 and 9.7%; so, it was an acceptable difference. The RSD of the slopes was calculated as a comparison of all slopes obtained during two weeks of storage stability studies and were calculated as SD values divided by the average value multiplied by 100%.

Moreover, two statistical tests were conducted to compare the obtained curves by comparing standard deviations. In the first one (F-Snedecor test), the calibration curves (for each ion separately) obtained during every day of the storage stability studies were compared to the day of fabrication. The F-values were lower than the critical F-value for most of the µPADs stored in the fridge which suggests that there was no difference between compared curves. The second test (Hartley test) allows for comparison of all obtained calibration curves from the whole period of storage stability tests (also by comparison of standard deviations). The obtained values of F_max_ were lower than the critical value (F_max0_ = 33.6) for all ions (F_max_ = 2.50, 7.33, and 7.67 for Fe^2+^, Zn^2+^, and Mn^2+^, respectively).

Finally, the developed trianalyte paper-based system was tested using artificial samples containing three ions in different concentrations. The obtained results are presented in Table 4. The obtained recoveries are good which may suggest that the developed system in the future might be used for real sample analysis.

## 3. Materials and Methods

### 3.1. Reagents

All reagents used were of analytical grade. Iron(III) chloride hexahydrate (product no. 31232), manganese chloride tetrahydrate (product no. 221279), zinc chloride (product no. 208086), Ferene S (product no. 82940), 4-(2-pyridylazo)resorcinol, PAR (product no. 323209), and xylenol orange disodium salt (XO, product no. 52097) were purchased from Sigma-Aldrich (Saint Louis, MO, USA). 1-(2-pyridylazo)-2-naphthol, PAN (product no. 146310050), was purchased form Thermo Scientific (Waltham, MA, USA). Ascorbic acid, hydrochloric acid, acetic acid, sodium acetate, sodium dihydrogen phosphate, disodium hydrogen phosphate, tetrahydrofuran (THF), and sodium hydrogen were bought from POCh (Gliwice, Poland). For all experiments, doubly distilled water was used.

Stock solutions of zinc(II) and manganese(II) ions were prepared in distilled water (to prepare standards, stock solutions were diluted with distilled water), whereas the stock solution of iron(III) ions was prepared in 0.01 mol/L HCl. Before each measurement, iron(II) standards were prepared by adding 0.375 mol/L ascorbic acid (daily freshly prepared) to iron(III) in a 1:1 volume ratio.

The stock solution of Ferene S (C = 90 mmol/L) was stable for a month. XO stock solution was prepared in 0.01 mol/L acetate buffer (pH = 4.4) and was stable for 2 months. Moreover, 1.2 g/L PAR solution was prepared in 0.1 mol/L phosphate buffer (pH = 12). On the other hand, PAN was prepared in THF (1.25 g/L of PAN). The optimal parameters of determination of all analytes are presented in Table 5.

### 3.2. Paper-Based Analytical Devices

For fabrication of the µPADs, Whatman filter paper no. 1 (product no. WHA1001185, Sigma-Aldrich, USA) was used throughout. The type of filter paper was chosen experimentally. All shapes and geometries were designed in CorelDraw software (version 2021.5) and printed using the solid-ink printer (Xerox ColorQube 8580, Xerox Corporation, Rochester, New York, NY, USA) and black wax ink (Cartridge-Free ColorQube Ink (Xerox Corporation, Rochester, New York, NY, USA, product no. 108R00966). Borders of all shapes are 1 mm thick. Printouts were heated in a laboratory oven (Alpina easyline EG 40, Alpina, Konin, Poland) with a temperature of 115 °C for 60 s as described elsewhere [24]. This practice ensures obtaining tight wax barriers.

The separate circle of 4 mm diameter was prepared in steps. At first, the filter paper was cut into strips of 3 × 1 cm and then dipped into the solution of PAN in THF. After THF evaporation, the paper strips were bathed in the solution of 0.3 mol/L NaOH for 1 min. Then, the strips were air dried. Afterwards, they were cut into circles of d = 4 mm using a punch.

The puzzle devices were prepared by glueing all elements (printed µPADs with two zones, 4 mm circle, and a narrow paper strip as a connection between the two main parts) on cold laminating pouches. The connector was 3 × 1 mm. The developed device is shown in Figure 5.

### 3.3. Detection

For the colorimetric detection, an office scanner (Epson L3151, Epson, Nagano, Japan) was applied. Filter paper pieces were placed on the cold laminating pouches to prevent the system components from moving. Detection was conducted after an experimentally chosen time—15 min after the standards/samples’ deposition.

The ImageJ software (version 1.54d) was applied to read the colors’ (red, green, and blue) intensities from the detection zones. Each detection zone has a defined “region of interest” (ROI) which was the whole area inside the hydrophobic wax barriers. The highest sensitivities were obtained for the following scanning options: photo mode with color restoration and tiff as the save format.

For selection of the appropriate computing method, the developed calculation sheet described and available for free elsewhere [15] was used. Among the 19 tested computing methods for each ion, were all of the most frequently used methods in the literature: single color intensities, average of sum of single color intensities, six versions of divisions (e.g., sum of all color intensities divided by the intensity of one, e.g., (R + G + B)/R, divisions one by one of the intensities, e.g., R/B, etc.), logarithms as log(I_0_/I_r_), signal difference between blank and signal after reaction as I_0_ − I_r_, sum of differences (∑I_0_ − I_r_), Euclidean distance, and A-RGB, which is expressed as −log((R × G × B)/(R_0_ × G_0_ × B_0_)). All computing methods, where possible, were used to analyze all color intensities. Not only sensitivities the were analyzed, but also, the differences in signals needed to distinguish similar values of concentrations were considered.

For Fe(II) ion detection, similar results were obtained for single red intensity, I_0_ − I_r_ for red intensity, Euclidean distance, and A-RGB. A bit better of a result was obtained when using the sum of signals differences (∑I_0_ − I_r_), but it was much more time consuming. For the purpose of this research, it was completely sufficient and much faster to use single red intensity (which was among the chosen best methods). In the case of Zn(II) ion detection, the best methods were the signal difference between blank and the signal after reaction as I_0_ − I_r_, as well as the Euclidean distance. For the measurements presented in this article, it is completely sufficient to use the signal differences. For Mn(II) ion detection, similar results (considering also the differentiation of similar concentrations) were obtained when using green intensity, I_0_ − I_r_ for green intensity, and Euclidean distance, and a slightly better results were obtained when using the sum of signals differences (∑I_0_ − I_r_). Taking into account the detection requirements in the presented work, it was completely sufficient to use single green intensity.

## 4. Conclusions

To sum up, in this work, a microfluidic paper-based analytical device dedicated to simultaneous detection of three analytes (Fe(II), Zn(II), and Mn(II) ions) has been developed. The system is characterized by good analytical parameters, among which better detection limit results were obtained than what has been presented in the literature. Using one zone for sample deposition and manganese(II) ion determination solved the problem of poor transport of Mn^2+^ ions through the filter paper matrix. Moreover, replacement of the more popular PAR dye, which is dedicated to many metal ions’ detection, with PAN, which is not water-dissolved, as well as preparing this zone separately, bypassed the problem with dye migration together with deposited standards/samples. Applying bathing in NaOH solutions improved linearity and sensitivity as well as the LOD of manganese detection because the color change was located only on the detection zone.

Moreover, each detection zone has another optimal pH for creating the complexes. To eliminate the risk of contamination of one detection zone environment by another’s, different angles of placing/designing detection zones were tested. In this work, it was proved that this solution works pretty well, and it might be useful also in future systems with many detection zones with different pHs.

## Figures and Tables

**Table 2:** Analytical parameters of the developed trianalyte µPAD for simultaneous determination of iron(II), manganese(II), and zinc(II) ions.

Analyte	LOD [mmol/L]	LOD [mg/L]	LOQ [mmol/L]	LOQ [mg/L]	Accuracy [%]	Precision [%]
Fe^2+^	0.0060	0.34	0.0199	1.11	89.8 ± 0.4	5.38 ± 0.90
Zn^2+^	0.0077	0.50	0.0123	0.80	94.6 ± 4.0	4.54 ± 0.74
Mn^2+^	0.0021	0.12	0.0065	0.36	103.3 ± 6.9	8.05 ± 0.72

**Table 3:** Comparison of analytical parameters of the developed µPAD with systems described elsewhere.

Analyte	Reagent	Detector	Analytical Range [mg/L]	LOD [mg/L]	Refs.
Fe^2+^	1,10-phenanthroline	smartphone	0.90–20.0	0.90	[22]
Zn^2+^	PAN, pH = 6	scanner	0.1–10	0.036	[23]
Zn^2+^	Zincon, pH = 9	smartphone	2.00–6.00	0.63	[2]
Mn^2+^	PAN, pH = 9	smartphone	0.20–1.00	0.11	[2]
Fe^2+^	Ferene S, pH = 2	scanner	0.34–22.4	0.34	This work
Zn^2+^	Xylenol orange, pH = 4.4		0.50–39.0	0.50	
Mn^2+^	PAN, pH = 12		0.12–8.25	0.12	

**Table 4:** Artificial samples analysis using the developed trianalyte paper-based device.

Sample No.	Analyte	Added [mmol/L]	Determined [mmol/L]	Recovery [%]
1	Fe^2+^	0.400	0.349 ± 0.001	87 ± 0.3
	Zn^2+^	0.800	0.817 ± 0.059	102 ± 7
	Mn^2+^	0.125	0.139 ± 0.010	111 ± 8
2	Fe^2+^	0.400	0.360 ± 0.026	90 ± 6
	Zn^2+^	0.800	0.735 ± 0.087	92 ± 10
	Mn^2+^	0.125	0.124 ± 0.002	99 ± 2
3	Fe^2+^	0.200	0.212 ± 0.210	106 ± 10
	Zn^2+^	0.500	0.544 ± 0.005	109 ± 1
	Mn^2+^	0.090	0.112 ± 0.001	124 ± 1
4	Fe^2+^	0.250	0.253 ± 0.012	101 ± 5
	Zn^2+^	0.500	0.466 ± 0.021	93 ± 4
	Mn^2+^	0.090	0.087 ± 0.001	96 ± 1

**Table 5:** The summary of the conditions of the µPADs’ preparation and measurements using them.

Parameter	Fe^2+^	Zn^2+^	Mn^2+^
**Standards concentrations**	0–1.0 mmol/L (in 0.01 mol/L HCl)	0–1.0 mmol/L (in water)	0–0.2 mmol/L (in water)
**Chromogenic reagent concentration**	90 mmol/L	5 mmol/L—2 layers	2.8 mmol/L
**Additional reagents/environment**	0.375 mol/L ascorbic acid/pH = 2	pH = 4.4	pH = 12
**Scanning options**	TIFF, with color restoration		
**RGB value**	R	G (as I_0_ − I_r_)	G
**Dye’s drying time**	15 min		
**The time gap: standard deposition detection**	15 min		
**Detection zone diameter**	5 mm	10 mm	4 mm

## Data Availability

All data generated or analyzed during this study are included in this published article and if needed may be made available in other forms upon request. Moreover, the raw data have been deposited at the RepOD repository, under the following deposition address: https://doi.org/10.18150/TG6PWL.

## Supplementary Materials

The following supporting information can be downloaded at: https://www.mdpi.com/article/10.3390/molecules29204805/s1. Figure S1. Optimization of iron ions detection. Calibration curves of iron ions obtained for each parameter selection: (**a**) and (**b**) concentration of Ferene S, (**c**) drying time of chromogenic reagent, (**d**) diameter of the detection zone.; Figure S2. Calibration curves of iron ions obtained in a simple microfluidic systems. The legend shows numbers referring to the length x width of channels connecting sample and detection zones. Figure S3. Comparison of results for different detection zone diameters, concentrations of xylenol orange and numbers of dye layers for zinc(II) ions determination: (**a**) and (**b**)—comparison of dye concentration at two diameters of detection zones, (**c**) and (**d**)—comparison of number of layers of dye at 10 mm diameter detection zone. Figure S4. Optimization of PAR concentration for Mn^2+^ ions detection in µPAD system. In the Table the obtained sensitivities in the range of 0–1.0 mmol/L of Mn^2+^ ions are presented. Figure S5. Calibration graphs (**a**) for Mn^2+^ ions, (**b**) for Fe^2+^ ions, obtained using the bianalyte flow system using standard solutions containing single ion (Fe or Mn) and their mixture. Figure S6. Calibration graphs for (**a**) Fe(II) ions, (**b**) Zn(II) ions, (**c**) Mn(II) ions obtained using the trianalyte flow system using standard solutions containing single ions and their mixture. In Tables behind the graphs the concentrations of not main ions are given; in all cases (**a**) Fe(II): 0, 0.1, 0.2, 0.3 mmol/L; in all cases in (**b**) Zn(II): 0, 0.2 0.4, 0.6 mmol/L; in all cases in (**c**) Mn(II): 0, 0.05 0.1, 0.15 mmol/L.
