# Supplementary material for: A Trianalyte µPAD for Simultaneous Determination of Iron, Zinc, and Manganese Ions
Source: Molecules. 2024 Oct 11;29(20):4805. doi: 10.3390/molecules29204805 (PMC11510364; doi:10.3390/molecules29204805)
Supplement: Supplementary file 1 [file molecules-29-04805-s001.zip › molecules-3206829-supplementary.pdf]

Supplementary Material

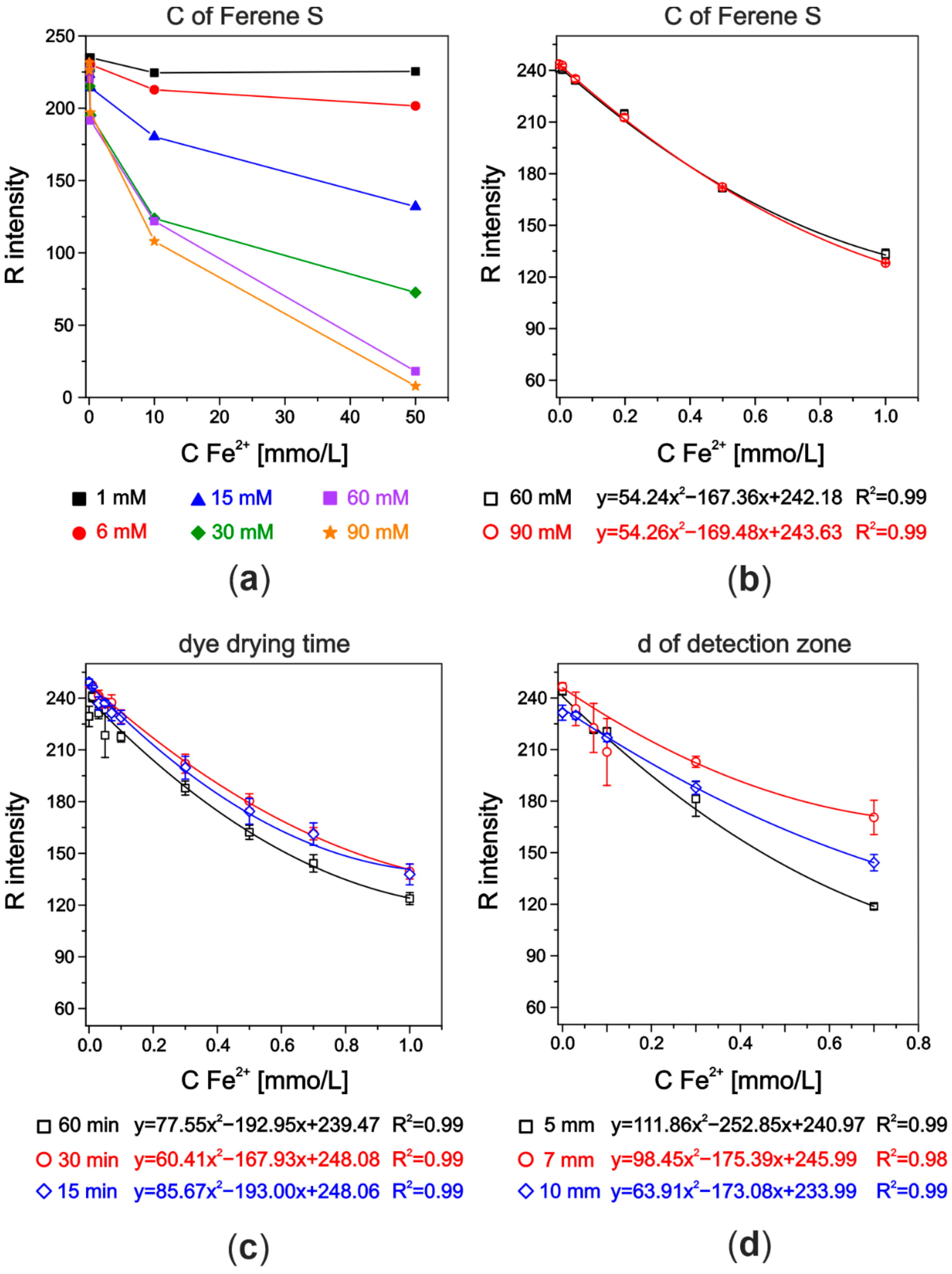

Figure S1. Optimization of iron ions detection. Calibration curves of iron ions obtained for each parameter selection: (**a**) and (**b**) concentration of Ferene S, (**c**) drying time of chromogenic reagent, (**d**) diameter of the detection zone.

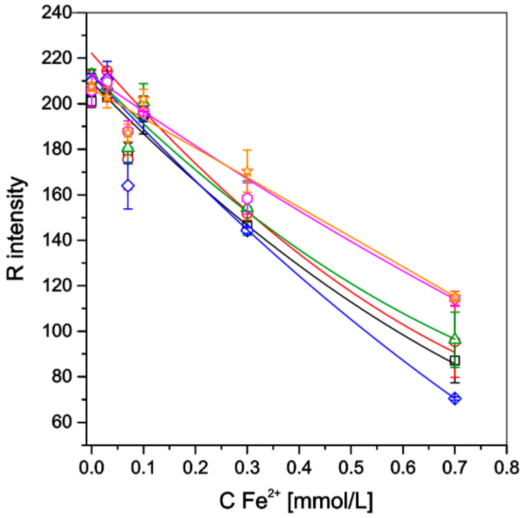

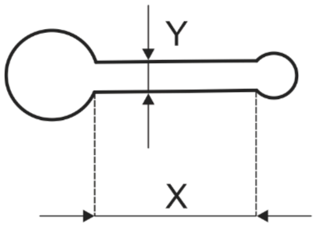

No.	X [mm]	Y [mm]	equation	R^2
□	20	3	$y=81.95x^2-234.27x+209.47$	0.94
◇	15	3	$y=59.19x^2-244.67x+212.69$	0.99
☆	10	3	$y=0.88x^2-129.85x+206.59$	0.99
○	20	2	$y=109.03x^2-264.17x+222.20$	0.98
△	15	2	$y=88.91x^2-228.77x+213.14$	0.94
⬡	10	2	$y=26.94x^2-159.18x+212.28$	0.98

Figure S2. Calibration curves of iron ions obtained in a simple microfluidic systems. The legend shows numbers referring to the length x width of channels connecting sample and detection zones.

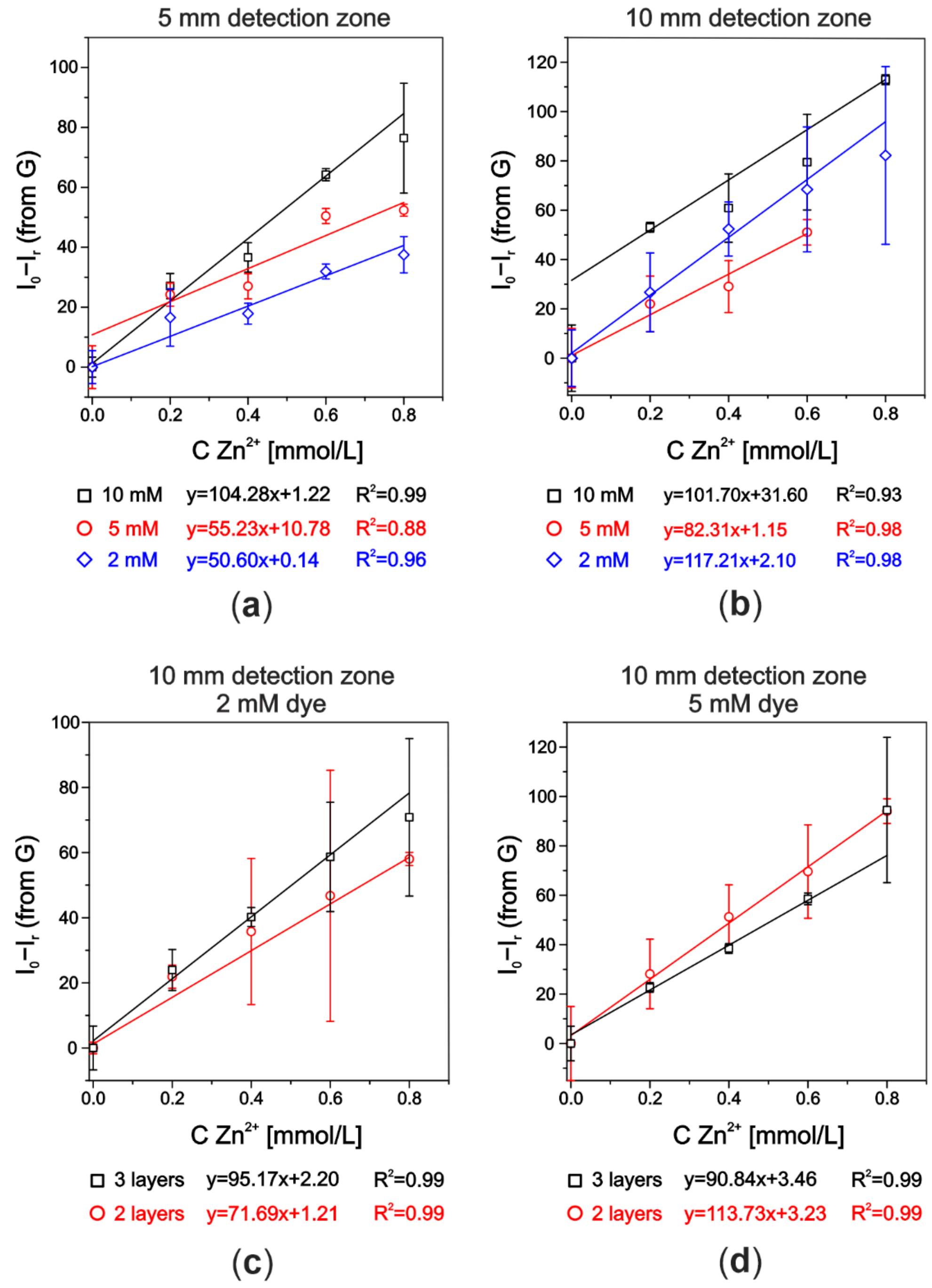

Figure S3. Comparison of results for different detection zone diameters, concentrations of xylenol orange and numbers of dye layers for zinc(II) ions determination: (**a**) and (**b**) – comparison of dye concentration at two diameters of detection zones, (**c**) and (**d**) – comparison of number of layers of dye at 10 mm diameter detection zone.

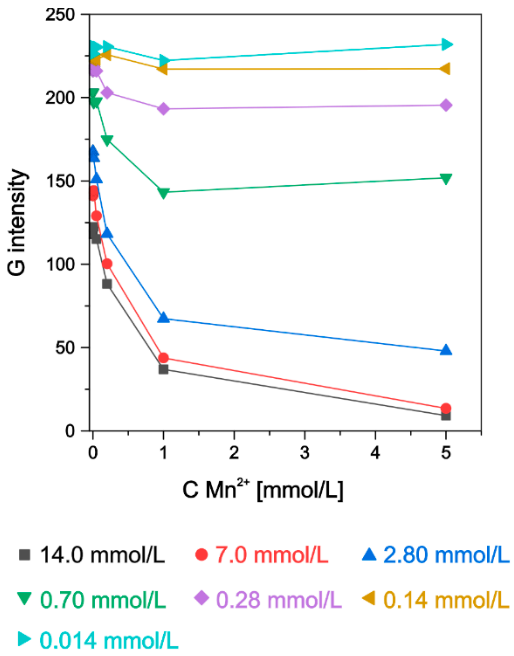

PAR concentration [mmol/L]	Sensitivity [L/mmol]
0.014	−6.94
0.14	−7.13
0.28	−23.36
0.70	−56.35
2.80	−95.83
7.0	−95.08
14.0	−83.23

Figure S4. Optimization of PAR concentration for Mn^{2+} ions detection in μPAD system. In the Table the obtained sensitivities in the range of 0-1.0 mmol/L of Mn^{2+} ions are presented.

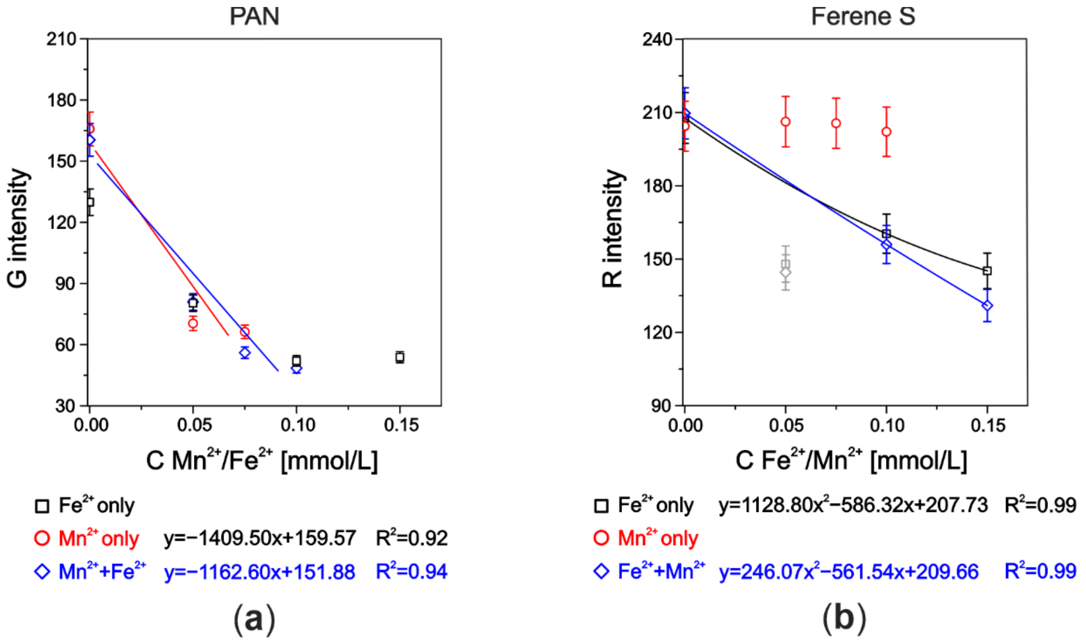

Figure S5. Calibration graphs (**a**) for Mn^{2+} ions, (**b**) for Fe^{2+} ions, obtained using the bianalyte flow system using standard solutions containing single ion (Fe or Mn) and their mixture.

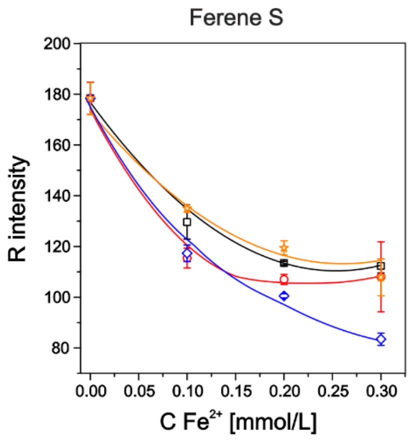

(**a**)

No.	Zn^{2+} [mmol/L]	Mn^{2+} [mmol/L]
□	0.2	0.1
○	0.2	0, 0.1, 0.15, 0.2
◇	0, 0.2, 0.4, 0.6	0.1
☆	0, 0.2, 0.4, 0.6	0, 0.1, 0.15, 0.2

□ $y=1030.69x^2-523.44x+176.86$ $R^2=0.99$
○ $y=1600.70x^2-699.61x+176.13$ $R^2=0.97$
◇ $y=1100.00x^2-631.57x+176.16$ $R^2=0.98$
☆ $y=881.71x^2-459.90x+173.18$ $R^2=0.97$

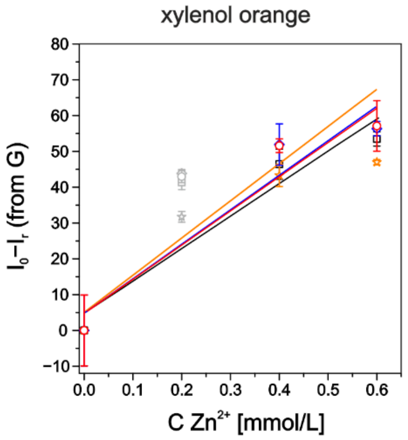

(**b**)

No.	Fe^{2+} [mmol/L]	Mn^{2+} [mmol/L]
□	0.1	0.1
◇	0.1	0, 0.1, 0.15, 0.2
○	0, 0.1, 0.2, 0.3	0.1
☆	0, 0.1, 0.2, 0.3	0, 0.1, 0.15, 0.2

□ $y=93.01x+2.31$ $R^2=0.96$
◇ $y=99.06x+3.08$ $R^2=0.93$
○ $y=100.00x+2.90$ $R^2=0.94$
☆ $y=108.23x+3.36$ $R^2=0.93$

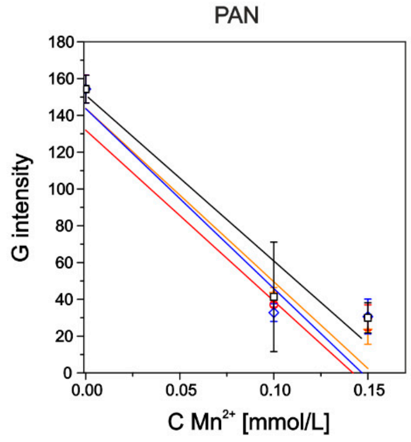

(**c**)

No.	Fe^{2+} [mmol/L]	Zn^{2+} [mmol/L]
□	0.1	0.2
◇	0.1	0, 0.2, 0.4, 0.6
○	0, 0.1, 0.2, 0.3	0.2
☆	0, 0.1, 0.2, 0.3	0, 0.2, 0.4, 0.6

□ $y=-834.76x+153.72$ $R^2=0.99$
◇ $y=-978.62x+143.71$ $R^2=0.89$
○ $y=-929.22x+132.00$ $R^2=0.83$
☆ $y=-942.70x+143.82$ $R^2=0.94$

Figure S6. Calibration graphs for (**a**) Fe(II) ions, (**b**) Zn(II) ions, (**c**) Mn(II) ions obtained using the trianalyte flow system using standard solutions containing single ions and their mixture. In Tables behind the graphs the concentrations of not main ions are given; in all cases (**a**) Fe(II): 0, 0.1, 0.2, 0.3 mmol/L; in all cases in (**b**) Zn(II): 0, 0.2 0.4, 0.6 mmol/L; in all cases in (**c**) Mn(II): 0, 0.05 0.1, 0.15 mmol/L.
